# Molecular mechanisms underlying progesterone-enhanced breast cancer cell migration

**DOI:** 10.1038/srep31509

**Published:** 2016-08-11

**Authors:** Hui-Chen Wang, Wen-Sen Lee

**Affiliations:** 1 Graduate Institute of Medical Sciences, College of Medicine, Taipei Medical University, Taipei 110, Taiwan; 2 Department of Physiology, School of Medicine, College of Medicine, Taipei Medical University, Taipei 110, Taiwan; 3 Cancer Research Center, Taipei Medical University Hospital, Taipei 110, Taiwan

## Abstract

Progesterone (P4) was demonstrated to inhibit migration in vascular smooth muscle cells (VSMCs), but to enhance migration in T47D breast cancer cells. To investigate the mechanism responsible for this switch in P4 action, we examined the signaling pathway responsible for the P4-induced migration enhancement in breast cancer cell lines, T47D and MCF-7. Here, we demonstrated that P4 activated the cSrc/AKT signaling pathway, subsequently inducing RSK1 activation, which in turn increased phosphorylation of p27 at T198 and formation of the p27pT198-RhoA complex in the cytosol, thereby preventing RhoA degradation, and eventually enhanced migration in T47D cells. These findings were confirmed in the P4-treated MCF-7. Comparing the P4-induced molecular events in between breast cancer cells and VSMCs, we found that P4 increased p27 phosphorylation at T198 in breast cancer cells through RSK1 activation, while P4 increased p27 phosphorlation at Ser10 in VSMCs through KIS activation. P27pT198 formed the complex with RhoA and prevented RhoA degradation in T47D cells, whereas p-p27Ser10 formed the complex with RhoA and caused RhoA degradation in VSMCs. The results of this study highlight the molecular mechanism underlying P4-enhanced breast cancer cell migration, and suggest that RSK1 activation is responsible for the P4-induced migration enhancement in breast cancer cells.

In developed countries, breast cancer is the most commonly occurring female cancer. Endogenous sex hormones are thought to influence the risk of developing of breast cancer^1^. Studies of the relationship between sex hormones and breast cancer in premenopausal women showed that the risk of breast cancer is positively associated with circulating concentrations of estrogens and androgens^2^,^3^,^4^. Experimental and epidemiological studies also suggest that estrogen and P4 are intimately linked to mammary carcinogenesis. The clinical findings from the Women’s Health Initiative and Million Women Study demonstrated that women taking progestin together with estrogen as part of hormone replacement experienced a greater breast cancer risk (larger tumor and higher grade) as compared with taking estrogen alone^5^,^6^. However, some clinical trials have also shown that combined estrogen and P4 hormone replacement therapy is associated with a very small increase in the risk of developing of breast cancer.

P4 is an ovarian steroid hormone. The central physiological roles of P4 in human reproduction include normal breast development during puberty, facilitation of implantation, maintenance of pregnancy, regulation of the signaling required for sexual behavior in the brain^7^,^8^. The actions of P4 are primarily mediated by binding to its high-affinity receptors, P4 receptor (PR)-A and/or PR-B isoforms. Co-treatment with P4 and estrogen are frequently prescribed for postmenopausal hormone replacement therapy. Estrogen has been indicated to be a potent breast mitogen, and inhibitors of the estrogen receptor, aromatases or estrogen-producing enzymes are effective first-line cancer therapies. Regarding the role of P4 in the development of breast cancer, P4 has been demonstrated to enhance proliferation^9^,^10^,^11^ and migration^12^ of breast cancer cells through extra-nuclear signaling pathways. Previously, it has been demonstrated that P4 drives PR-A to interact with the G protein Gα13, whereas medroxyprogesterone acetate drives PR to interact with cSrc and to activate PI3K, leading to the activation of RhoA/ROCK-2 in breast cancer cell lines^12^. However, the signaling pathway underlying P4-induced migration enhancement in breast cancer cells is still not fully elucidated.

In the present investigation, we used *in vitro* system to study how P4 affect the migration of T47D and MCF-7 breast cancer cell lines. These experimental findings reported below highlight certain molecular mechanisms underlying P4-induced migration enhancement in breast cancer cells. Only when the molecular mechanism underlying P4-induced migration enhancement in breast cancer cells is fully understood can we begin to design a strategy for treating the P4-enhanced breast cancer cell migration.

## Results

### Roles of p27 up-regulation and RhoA activation in the P4-induced migration enhancement in T47D cells

**Figure 1:**
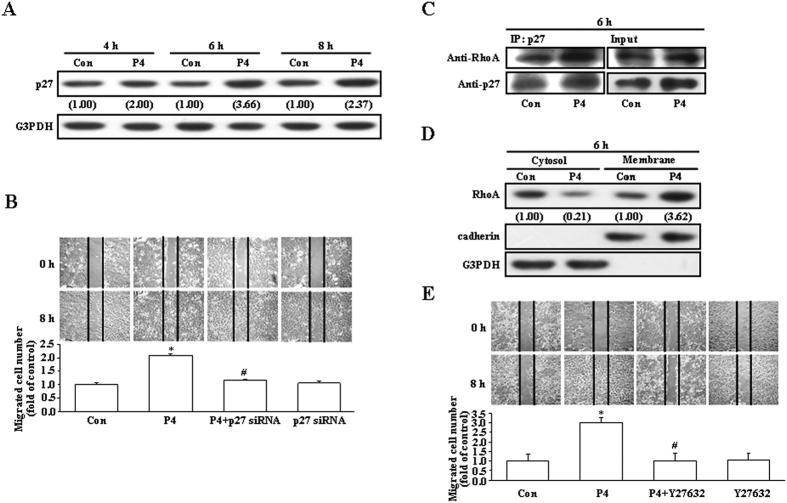
Involvement of p27 and RhoA in the P4-induced enhanced migration in T47D cells. (**A**) Treatment with P4 (50 nM) for 4–8 h increased the level of p27 protein in T47D cells. (**B**) Knock-down of p27 abolished the P4-induced migration enhancement in T47D cells. P4 increased formation of the p27-RhoA complex (**C**) and membrane translocation of RhoA from the cytosol (**D**). (**E**) Pre-treatment with a ROCK inhibitor, Y27632 (5 μM), prevented the P4-induced migration enhancement in T47D cells. For Western blot analyses, data are representative of 2 independent experiments with similar results. Values shown in parentheses represent the quantified results adjusted with G3PDH (**A**) or with G3PDH and cadherin for the cytosol and the membrane, respectively (**D**) and expressed as ratio over its own control. cadherin and G3PDH were used as a membranous and cytosolic protein marker, respectively, to confirm the purities of isolation and to verify equivalent sample loading. The gels have been run in the same experimental conditions and the cropped blots were shown. The entire gel pictures of 1C were shown in the Supplemental Fig. 1. In (**B**,**E**), values represent the means±s.e.mean. (n = 3). *P < 0.05 different from control group. ^#^*P* < 0.05 different from P4-treated group. Con, control; IB, immunoblotting; IP, immunoprecipitation; siRNA, small interfering RNA.

Previously, we demonstrated that inactivation of RhoA mediated by up-regulation of p27 is involved in the P4-induced migration inhibition in rat aortic smooth muscle cells (RASMCs)^13^. In the present study, we investigated whether up-regulation of p27 is involved in the P4-induced migration enhancement in breast cancer cells. Initially, we used T47D breast cancer cell line to address this issue. As shown in Fig. 1A, treatment with P4 (50 nM) for 4–8 h increased the levels of p27 protein in T47D cells. However, pre-transfection with p27 siRNA significantly reduced the P4-induced migration enhancement in T47D cells (Fig. 1B), suggesting that up-regulation of p27 contributed to the P4-induced migration enhancement in T47D cells. Since it has been indicated that RhoA plays an important role in regulating cell motility, we next examined the involvement of RhoA activation in the P4-enhanced migration in T47D cells. Treatment with P4 for 6 h increased formation of the p27-RhoA complex (Fig. 1C) and membrane translocation of RhoA from the cytosol (Fig. 1D). Moreover, pre-treatment with Y27632 (5 μM), a ROCK inhibitor (a kinase associated with RhoA for transducing RhoA signaling), prevented the P4-induced migration enhancement in T47D cells (Fig. 1E). These data suggest that RhoA activation is also involved in the P4-enhanced migration in T47D cells.

### Role of RSK1 in the P4-induced migration enhancement in T47D cells

**Figure 2:**
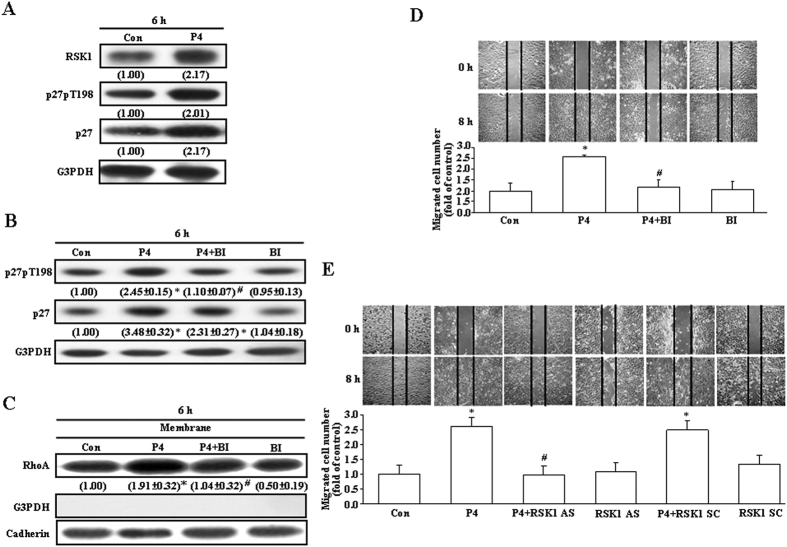
Involvement of RSK1 activation in the P4-induced migration enhancement in T47D cells. (**A**) P4 (50 nM) treatment increased the levels of RSK1, p27 and p27pT198 protein in T47D cells. Pre-treatment with a RSK1 inhibitor, BI-D1870 (31 nM), prevented P4-induced increases in p27 phosphorylation at T198 (**B**), membrane translocation of RhoA (**C**), and migration enhancement (**D**). (**E**) Pre-treatment of the cell with RSK1 antisense oligonucleotide prevented the P4-induced migration enhancement in T47D cells. For Western blot analyses, data are representative of 2 (**A**) or 3 (**B**,**C**) independent experiments with similar results. Values shown in parentheses represent the quantified results adjusted with G3PDH (**A,B**) or cadherin (**C**) and expressed as ratio over its own control. Caherin and G3PDH were used as a membrane and cytosolic protein marker, respectively, to confirm the purities of isolation and to verify equivalent sample loading. In (**B**–**E**), values represent the means±s.e.mean. (n = 3). *P < 0.05 different from control group. ^#^*P* < 0.05 different from P4-treated group. AS, antisense; BI, BI-D1870; Con, control; SC, scramble.

The phosphorylation site of p27 might affect its subcellular localization and functions. It has been indicated that phosphorylation of p27 at ser10 might increase its translocation from the nucleus to the cytosol, subsequently enhancing formation of the p-p27ser10-RhoA complex, and then cause migration inhibition^13^, whereas phosphorylation of p27 at T198 might increase formation of the p27pT198-RhoA complex^14^, prevent ubiquitin-dependent degradation of p27^15^, and promote cell motility^14^. RSK1 activation can drive p27 phosphorylation at T198 to increase cell motility^14^. Accordingly, we examined the involvement of RSK1 activation in p27 phosphorylation at T198 and cell motility in T47D cells. Treatment with P4 (50 nM) increased the levels of p27, RSK1 and p27pT198 protein (Fig. 2A) in T47D cells. Pre-treatment with BI-D1870 (31 nM), a RSK1 inhibitor, prevented the P4-increased level of p27pT198 protein (Fig. 2B), membrane translocation of RhoA (Fig. 2C), and migration (Fig. 2D), suggesting that RSK1 activation might play an important role in the P4-enhanced migration in T47D cells. To confirm the involvement of RSK1 in the P4-induced migration enhancement in T47D cells, the expression of RSK1 was knocked-down by antisense oligonucleotide. As shown in Fig. 2E, the P4-induced migration enhancement in T47D cells was abolished by pre-treatment the cell with the RSK1 antisense oligonucleotide, but not with the scramble oligonucleotide.

### Involvement of cSrc activation in the P4-induced up-regulation of p27 protein and migration enhancement in T47D cells

**Figure 3:**
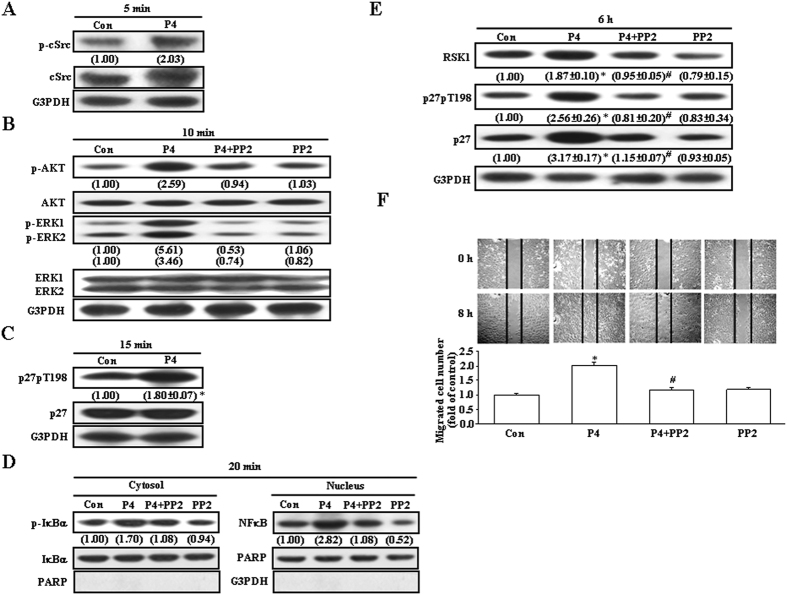
Role of the cSrc-mediated pathway in the P4-induced migration enhancement in T47D cells. (**A**) Treatment with P4 (50 nM) for 5 min induced cSrc activation in T47D cells. (**B**) Treatment with P4 (50 nM) for 10 min induced activations of AKT and ERK1/2, and these effects were abolished by pre-treatment of the cell with a cSrc antagonist, PP2 (200 nM). (**C**) Treatment with P4 (50 nM) for 15 min increased the level of p27pT198. (**D**) Treatment with P4 (50 nM) for 20 min induced IκBα activation and NFκB nuclear translocation, and these effects were abolished by pre-treatment of the cell with PP2. (**E**) Treatment with P4 (50 nM) for 6 h induced increases in the levels of RSK1, p27 and p27pT198 protein. (**F**) The P4 (50 nM)-induced migration enhancement was abolished by pre-treatment of the cell with PP2. Western blot data are representative of 2 (**A**,**B**,**D**) or 3 (**C**,**E**) independent experiments with similar results. Values shown in parentheses represent the quantified results adjusted with total IκBα and PARP for p-IκBα in the cytosol and NFκB in the nucleus, respectively (**D**), with their own total protein (**A**–**C**), or with G3PDH (**E**), and are expressed as ratio over its own control. In (**C**,**E**,**F**), values represent the means±s.e.mean. (n = 3). *P < 0.05 different from control group. ^#^*P* < 0.05 different from P4-treated group. Con, control.

Previously, we demonstrated that P4 might inhibit RASMCs migration through up-regulation of p27 mediated by activating the cSrc/AKT/ERK/p38 signaling pathway^16^. Accordingly, we examined whether this signaling pathway is also involved in the P4-induced up-regulation of p27 and migration enhancement in T47D cells. Treatment with P4 (50 nM) for 5–15 min activated cSrc (Fig. 3A), AKT and ERK1/2 (Fig. 3B), and induced phosphorylation of p27 at T198 (Fig. 3C). Pre-treatment with PP2 (200 nM), a cSrc inhibitor, abolished activations of AKT (Fig. 3B), ERK1/2 (Fig. 3B) and IκBα (Fig. 3D, left panel), and increases of the nucleus translocation of NFκB (Fig. 3D, right panel). The increased levels of RSK1, p27 and p27pT198 protein (Fig. 3E) and migration enhancement (Fig. 3F) in T47D cells induced by P4 treatment were also abolished by pre-treatment with PP2. These data suggest that P4-induced increases in the levels of RSK1, p27 and p27pT198 protein were mediated through a cSrc-mediated pathway.

### Role of AKT in the P4-induced phosphorylation of p27 at T198 and migration enhancement in T47D cells

**Figure 4:**
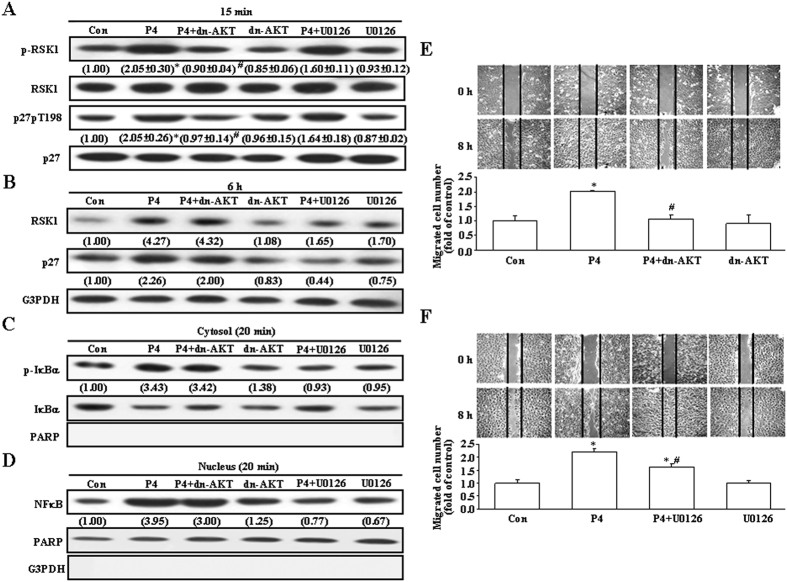
Roles of AKT and ERK1/2 activation in the P4-induced migration enhancement in T47D cells. (**A**) Treatment with P4 (50 nM) for 15 min increased the levels of p-RSK1 and p27pT198, and these effects were abolished by pre-treatment of the cell with dn-AKT, but not U0126 (1 μM). (**B**) Treatment with P4 (50 nM) for 6 h increased the levels of RSK1 and p27 protein, and these effects were abolished by pre-treatment of the cell with U0126, but not dn-AKT. Treatment with P4 (50 nM) for 20 min induced IκBα activation (**C**) and NFκB nuclear translocation (**D**), and these effects were abolished by pre-treatment of the cell with U0126, but not dn-AKT. Western blot data are representative of 2 (**B**–**D**) or 3 (**A**) independent experiments with similar results. Values shown in parentheses represent the quantified results adjusted with their own total protein (**A**,**C**), G3PDH (**B**), or PARP (**D**). PARP and G3PDH were used as a nucleus and cytosolic protein marker, respectively, to confirm the purities of isolation and to verify equivalent sample loading. The P4 (50 nM)-induced migration enhancement was abolished by pre-transfection of cells with dn-AKT (**E**) or partially reduced by pre-treatment with 1 μM of U0126 (**F**) in T47D cells. In (**A**,**E**,**F**), values represent the means±s.e.mean. (n = 3). *P < 0.05 different from control group. ^#^*P* < 0.05 different from P4-treated group. Con, control; dn-AKT, dominant negative AKT.

It has been indicated that RSK1 is an effector of both Ras/MEK/MAPK and PI3K/PDK1/AKT pathways. Therefore, we further examined whether activations of PI3K and ERK are involved in the P4-induced RSK1 activation. Treatment with P4 for 15 min did not affect the levels of RSK1 and p27 protein, but increased the levels of p-RSK1 and p27pT198, and these effects were prevented by pre-transfection with dn-AKT, but not by pre-treatment with U0126 (1 μM), an ERK inhibitor (Fig. 4A). However, treatment with P4 for 6 h increased the levels of both total and phosphorylated RSK1 and p27 protein. As shown in Fig. 4B, pre-treatment with U0126, but not with dn-AKT, significantly reduced P4-induced increases in the levels of RSK1 and p27 protein, whereas pre-treatment with dn-AKT, but not with U0126, significantly reduced P4-induced increases in the levels of p-RSK1 and p27pT198 protein. These findings suggest that P4 induced RSK1 activation mainly through activating the AKT-mediated pathway and increases in the levels of RSK1 and p27 protein mainly through activating the ERK1/2-mediated pathway. To further confirm this notion, the following experiments were conducted. Treatment with P4 for 20 min induced IκBα activation (Fig. 4C) and nuclear translocation of NFκB (Fig. 4D), and these effects were prevented by pre-treatment with U0126, but not with dn-AKT. Moreover, the P4-induced migration enhancement was completely abolished by pre-transfection with dn-AKT (Fig. 4E) and reduced for only 25% by pre-treatment with U0126 (Fig. 4F).

### Requirement of new protein synthesis in the P4-induced RSK1 activation, phosphorylation of p27 at T198, and migration enhancement in T47D cells

**Figure 5:**
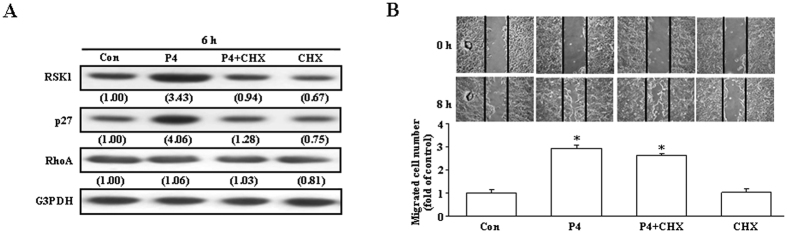
Requirement of new protein synthesis in the P4-induced migration enhancement in T47D cells. (**A**) Pre-treatment with an inhibitor of protein synthesis, cycloheximide (4 μM), prevented the P4 (50 nM)-induced increases of RSK1 and p27 protein. Western blot data are representative of 2 independent experiments with similar results. Values shown in parentheses represent the quantified results adjusted with G3PDH. (**B**) Pre-treatment with cycloheximide did not significantly affect the P4-induced migration enhancement. Values represent the means±s.e.mean. (n = 3). *P < 0.05 different from control group. CHX, cycloheximide; Con, control.

We further examined whether increases of protein syntheses are required for the P4-induced migration enhancement. Pre-treatment with, cycloheximide (4 μM), an inhibitor of protein biosynthesis, prevented the P4-increased the levels of RSK1 and p27 protein (Fig. 5A), but did not significantly affect the P4-induced migration enhancement (Fig. 5B), suggesting that new protein synthesis is not essential for the P4-induced migration enhancement.

### Involvement of the cSrc/AKT/RSK1-mediated signaling pathway in the P4-induced p27 phosphoryation at T198 and migration enhancement in MCF-7 cells

**Figure 6:**
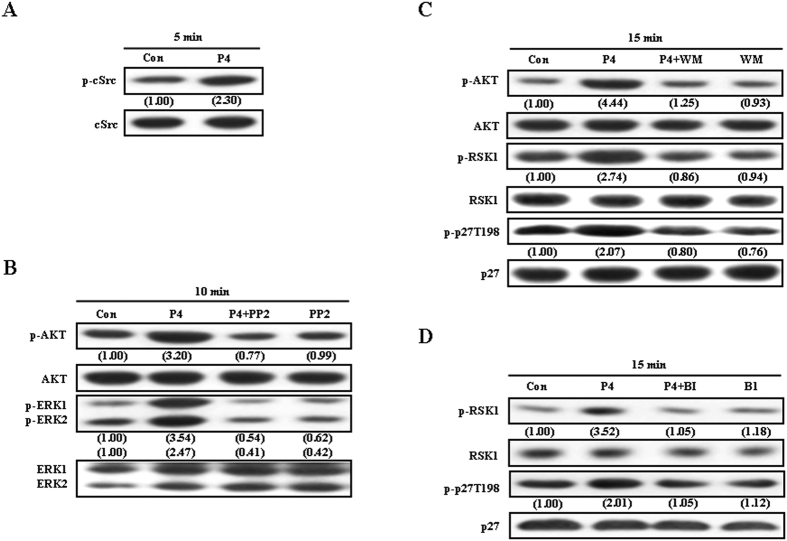
Effects of P4 on the activation of the cSrc/AKT/RSK1 signaling pathway and p27 phosphorylation at T198 in MCF-7 cells. (**A**) Treatment with P4 (50 nM) for 5 min induced cSrc activation in MCF-7 cells. (**B**) Pre-treatment with PP2 (100 nM) abolished the P4-induced activations in AKT and ERK1/2. (**C**) Pre-treatment with wortaminnin (100 nM) abolished P4-induced increases of the levels of p-RSK1 and p-p27T198 protein. (**D**) The P4-induced phosphorylation of p27 at T198 in MCF-7 was abolished by pre-treatment with BI-D1870. Western blot data are representative of 2 independent experiments with similar results. Values shown in parentheses represent the quantified results adjusted with G3PDH. Con, control; BI, BI-D1870; WM, wortmannin.

**Figure 7:**
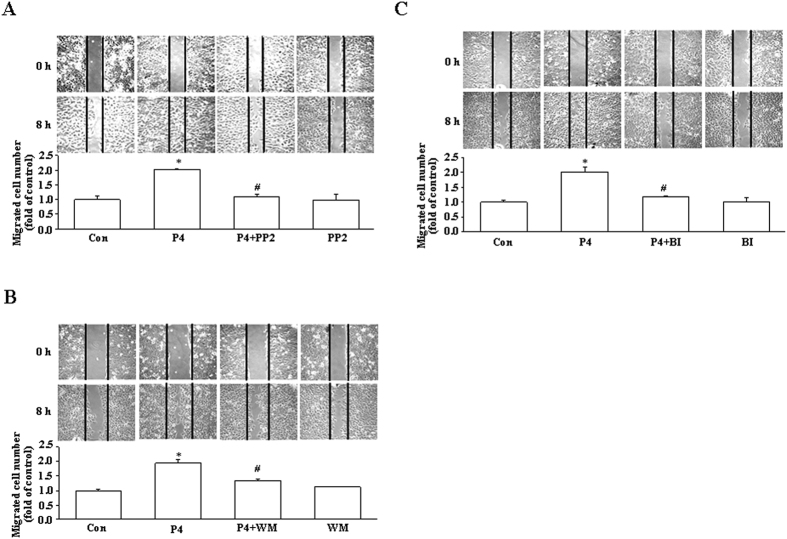
Involvement of cSrc/AKT/RSK1 activation in the P4-induced migration enhancement in MCF-7 cells. The P4 (50 nM)-induced migration enhancement in MCF-7 cells was abolished by pre-treatment with 200 nM of PP2 (**A**), 100 nM of wortmannin (**B**) or 31 nM of BI-D1870 (**C**). Values represent the means±s.e.mean. (n = 3). *P < 0.05 different from control group. ^#^*P* < 0.05 different from P4-treated group. Con, control; BI, BI-D1870; WM, wortmannin.

To examine whether the signaling pathway involved in the P4-induced migration enhancement in T47D cells was also observed in other breast cancer cell lines, the effect of P4 on the migration of MCF-7 breast cancer cells was studied. As shown in Fig. 6A, treatment with P4 (50 nM) for 5 min induced cSrc activation in MCF-7 cells. Pre-treatment with PP2 (200 nM) abolished P4-induced activations in AKT and ERK1/2 (Fig. 6B), whereas pre-treatment with wortmannin (100 nM), a PI3K inhibitor, abolished P4-induced increases of the levels of p-RSK1 and p27pT198 protein (Fig. 6C). The P4-induced phosphorylation of p27 at T198 in MCF-7 was abolished by pre-treatment with BI-D1870 (31 nM). Moreover, the P4-induced migration enhancement in MCF-7 was abolished by pre-treatment with PP2 (Fig. 7A), wortmannin (Fig. 7B) or BI-D1870 (Fig. 7C).

## Discussion

P4 was previously shown to promote T47D movement via actin cytoskeleton remodeling mediated by the RhoA/ROCK-2 cascade-triggered moesin activation^12^. However, the signaling pathway involved in the cSrc/PI3K-mediated activation of the RhoA/ROCK2 cascade is still unclear. In the present study, we further investigated the molecular mechanism underlying the P4-induced migration enhancement in breast cancer cells. Our results showed that P4 stimulated the cSrc/AKT signaling pathway to active RSK1, which in turn induced p27 phosphorylation at T198, subsequently increasing formation of the p27-RhoA complex and causing membrane translocation of RhoA, and finally enhanced migration in breast cancer cells. To our knowledge, this is the first demonstration that the RSK1-induced activation of RSK1 and phosphorylation of p27 at T198 are responsible for the P4-induced migration enhancement in breast cancer cell lines.

P27, an inhibitor of cyclin/cyclin-dependent kinase (CDK) complexes, can bind with cyclin-CDK complexes and cause inactivation of specific CDK enzymes required for cell division, and thereby arrest the cell cycle in the G1 phase^17^,^18^. It has been shown that targeted disruption of the CDK-inhibitory domain of mouse p27 gene results in enhanced growth of mice, multiple organ hyperplasia, and a predisposition to tumors^19^,^20^,^21^. Therefore, p27 has been considered as a tumor suppressor because of its function as a negative regulator of the cell cycle^22^,^23^. In addition to the function in regulating cell cycle activity, p27 has also been indicated to play additional roles outside of the nucleus^24^ and to be involved in regulating cancer cell differentiation^25^,^26^ and apoptosis^27^ and cell migration^28^.

The activity of p27 is controlled by its concentration, distribution among different cellular complexes, and subcellular localization^29^,^30^. P27 contains nuclear export signal (NES)^31^ and nuclear localization signal (NLS)^32^. In normal cells, p27 is largely localized in the nucleus and regulates cell cycle activity. In contrast, many human cancers exhibit cytoplasmic p27 localization^22^. It has been demonstrated that AKT and serum and glucocorticoid-regulated kinase 1 (SGK1) can phosphorylate p27NLS at T157^25^,^33^,^34^,^35^, and their constitutive activation localizes p27 to the cytoplasm by impairing its import into the nucleus^25^. In RASMCs, we previously demonstrated that P4 induced phosphorylation of p27 at serine 10 (Ser10) in the nucleus, subsequently enhancing p27 export from the nucleus to the cytosol, which in turn increased formation of the p27-RhoA complex, and finally caused p27 degradation through the ubiquitin-proteasome pathway^13^. In the present study, our data showed that P4 increased the level of p27pT198 protein (Fig. 2A) and formation of the p27-RhoA complex (Fig. 1C) in T47D, but did not affect the level of KIS protein (data not shown) in T47D cells. Threonine T198 in the c-terminal of p27 is a phosphorylation site, which controls several important aspects of p27 function including binding to cyclin-CDK complexes, subcellular localization and protein stability^15^. It was previously reported that phosphorylation of p27 at T198 increases formation of the p27-RhoA complex^14^ and prevents ubiquitin-dependent degradation of p27^15^. Our data also showed that knock-down of p27 or RSK1 expression, blockade of the RSK1 activity, or interruption of the RhoA-mediated pathway (Fig. 1E) abolished the P4-induced enhanced migration in T47D cells.

RSK1, an effector of both Ras/MEK/MAPK and PI3K/PDK1 pathways, can induce p27 phosphorylation at T198 to increase formation of the RhoA-p27 complex, p27 stability and cell motility^14^. In the present study, we showed that P4 activated RSK1 in T47D cells (Fig. 4A). Our data suggest that the P4-induced activation of RSK1 through a direct activation of the cSrc/AKT-mediated pathway. Pre-treatment with the RSK1 inhibitor, BI-D1870, prevented the P4-induced phosphorylation of p27 at T198 and migration enhancement in both T47D and MCF-7 cells, suggesting that RSK1 activation plays an important role in the P4-induced migration enhancement in breast cancer cells. The involvement of RSK1 in the P4-induced migration enhancement in breast cancer cells was further confirmed by the evidence showing that knock-down of RSK1 expression abolished the P4-induced migration enhancement. P4 also induced up-regulations of RSK1 and p27 through activating the cSrc/ERK1/2/NFκB-mediated pathway. However, pre-treatment with cycloheximide, an inhibitor of protein synthesis, which blocked the P4-induced up-regulations of RSK1 and p27 (Fig. 5A), did not cause any significant reduction in the P4-induced migration enhancement in T47D cells (Fig. 5B), suggesting that new protein synthesis might not be required for the P4-induced migration enhancement.

P4-induced activation of cSrc is mediated through a non-genomic action of P4^36^. This rapid action of P4 in the cytosol and consequent activation of cSrc has also been implicated in the regulation of cell cycle and migration^37^,^38^. Further indication that cSrc activation is involved in P4-induced activations of RSK1 and migration enhancement was evidenced by failure of these effects of P4 to occur in the PP2-treated T47D and MCF-7 cells. Taken together, these results suggest that activation of the cSrc-mediated signaling pathway might be involved in the P4-induced migration enhancement in breast cancer cells.

**Figure 8:**
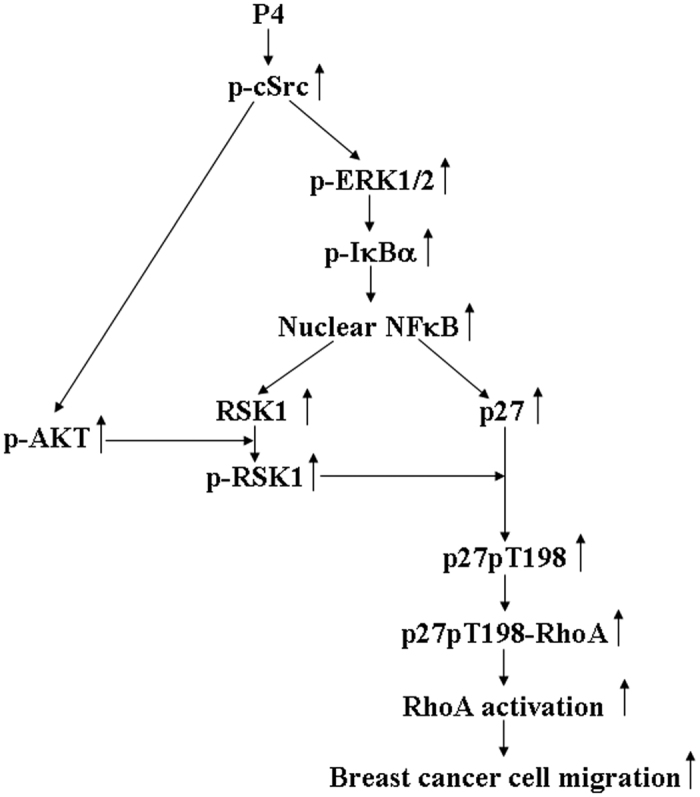
Model for P4-induced migration enhancement in breast cancer cells. P4 activated the cSrc/AKT signaling pathway, which in turn induced activation of RSK1, subsequently causing p27 phosphorylation at T198 and increasing formation of the p27-RhoA complex, and eventually enhanced migration of breast cancer cells. P4 also activated the cSrc/ERK1/2 signaling pathway to induce IκBα activation and NFκB nuclear translocation, which in turn up-regulated the expression of RSK1 and p27. The increased expression of RSK1 and p27 are not required, but might have some degree of contributions, for the P4-induced migration enhancement in breast cancer cells.

In conclusion, this study provides evidence that P4 induced RSK1 activation mediated by the cSrc/AKT signaling pathway, subsequently causing phosphorylation of p27 at T198, which in turn increased formation of the p27-RhoA complex and RhoA activation, and finally enhanced migration in breast cancer cells. P4 also increased the levels of p27 and RSK1 protein through activating the cSrc/ERK1/2 signaling pathway in T47D cells. Although increased expressions of RSK1 and p27 are not required for the P4-induced migration enhancement in T47D cells, it might have some contributions in the P4-regulated T47D cell migration. Based on the results from the present study, we propose a model of the molecular mechanism underlying the P4-induced migration enhancement in breast cancer cells as shown in Fig. 8. The findings from the present study suggest that activation of RSK1 is the key factor responsible for the P4-induced migration enhancement in breast cancer cells.

## Methods

### Cell Cultures

T47D and MCF-7 cell lines established from the pleural effusion of a ductal carcinoma of the breast were purchased from the American Type Culture Collection/Bioresource Collection and Research Center (BCRC) (Taiwan). These cells have performed STR-PCR profile at BCRC. The cells were grown in DMEM (GIBCO, Grand Island, NY) supplemented with 10% fetal bovine serum (FBS; GIBCO), penicillin (100 U/mL), and streptomycin (100 μg/mL, GIBCO) in a humidified 37 °C and 5% CO_2_ incubator, and the media was switched to DMEM supplemented with 10% charcoal-stripped fetal bovine serum at 24 h prior to and during P4 or vehicle (0.05% DMSO) treatment. For the experiments, T47D and MCF-7 cells were treated with either P4 (50 nM) in 0.05% DMSO (Sigma-Aldrich, St. Louis, MO) for the indicated times or 0.05% DMSO (control).

### Reagents

Antibodies against p-AKT, AKT, cadherin, p-ERK1/2, PARP, p65, p-IκBα, IκBα, p-RSK1, RSK1, p-p27T198 or RhoA and BI-D1870 were purchased from Santa Cruz Biotechnology, Inc. (Santa Cruz, CA). Anti-α-tubulin, anti-ERK1/2, and anti-p27 antibody and cycloheximide were purchased from Sigma-Aldrich (St. Louis, MO). Anti-G3PDH antibody was purchased from GeneTex (Hsinchu, Taiwan). Anti-cSrc antibody was purchased from Abcam (Cambridge, MA). Anti-p-cSrc antibody was purchased from Cell Signaling Technology Inc. (Beverly, MA). PP2 was purchased from A. G. Scientific, Inc. (San Diego, CA). U0126 was purchased from Cayman Chemical (Ann Arbor, MI).

### Wound healing assay

Wound healing assay was performed as previously described^16^,^39^. Briefly, cells were grown in 24-well plates. White tips for 10 μL micropipette were used to scalp a “wound” in a cell monolayer. The images captured at the beginning were compared to the images after 8 h in a humidified 37 °C, CO_2_ incubator. The cells migrating to close the wound were counted to quantify the migration rate of the cells.

### Western blot analysis

To determine the protein levels in breast cancer cells, total proteins were extracted and Western blot analyses were performed as described previously^16^. Electrophoresis was carried out using a 12% SDS-polyacrylamide gel (50 μg protein per lane). Separated proteins were transferred onto PVDF membranes, treated with 1% BSA/0.02% NaNO_3_ to block the non-specific IgGs, incubated for 1 h with specific primary antibody (0.2 μg/mL), and then incubated with second antibody (Jackson ImmunoResearch Laboratories, West Grove, PA) linked to horse radish peroxidase (1:10,000) for 1 h. Subsequently, the blot was developed using the ECL (enhanced chemiluminescence) system (GE, Healthcare, NJ). The intensity of each band was quantified by densitometry analysis using Image Pro-Plus 4.5 Software.

### Subcellular fractionation

To examine the membrane translocation of RhoA, breast cancer cells were washed with ice-cold phosphate-buffered saline (PBS) and re-suspended in hypotonic buffer (20 mM Tris-HCl, pH 7.5, 5 mM EGTA, 2 mM EDTA, 1 mM NaVO_3_, 1 mM DTT, 10 mM NaF, 10 mM Na_2_H_2_P_2_O_7_) plus protease inhibitor cocktail (Sigma-Aldrich). After being frozen at −80 °C for 2 h, the cell lysates were disrupted by passing through a 3/10 mL BD insulin syringe and 30-gauge needle for 120 times, and then incubated on ice with repeated vortex mixing for 6 times (5 min each time). The supernatant was collected as the cytosolic fraction after being centrifuged at 14,000 g for 10 min at 4 °C. Pellets were washed with ice-cold PBS for 2 times, and then lysed in the extract buffer (50 mM HEPES, pH 7.0, 250 mM NaCl, 2.5 mM EDTA, 1% NP-40, 5% Glycerol, 1 mM NaVO_3_, 10 mM NaF, 10 mM Na_2_H_2_P_2_O_7_) plus protease inhibitor cocktail (Sigma-Aldrich) on ice with repeated vortex mixing for 9 times (5 min in each), and then centrifuged at 14,000 g for 30 min at 4 ***°***C. The supernatant was collected as the particulate (membrane) fraction.

### Nuclear extraction

To examine the effect of P4 on nuclear translocation of NFκB (p65), the NE-PER nuclear and cytoplasmic extraction reagents (Thermo Fisher Scientific, Rockford, IL) were used and the extraction was performed as previously described^38^.

### Cell transfection

For transient transfection of the indicated constructs into T47D cells, jetPEI-T47D transfection reagent (Polyplus Transfection, Bioparc, France) was used and the transfection was performed as previously described^40^.

### Small interfering RNA (siRNA) knockdown assay

Expression of p27 was knocked-down in T47D with at least three independent small interfering RNAs (siRNAs). The target sequences of p27 mRNA were selected to suppress *p27* gene expression. Non-target sequences of each siRNA (NT-siRNA) were used as controls^41^. After BLAST analysis to verify that there were no significant sequence homologies with other human genes, the selected sequences were inserted into *Bgl*II/*Hin*dIII-digested pSUPER vectors to generate the pSUPER-Si p27 (p27-Si) and pSUPER-non-target p27 (p27-NT). The cells were transfected with either combined three anti-sense siRNAs or non-target RNA (control). Three different anti-sense siRNAs targeted against different parts of the p27 sequence are listed below: Si-1: 5′-GATCCCCGGAAGCGACCTGCAACCGATTCAAGAGATCGGTTGCAGGTCGCTTCCTTTTTA-3′; Si-2: 5′-GATCCCCACCCGGGAGAAAGATGTCATTCAAGAGATGACATCTTTCTCCCGGGTTTTTTA-3′; Si-3: 5′-GATCCCCGAAGCGACCTGCAACCGACTTCAAGAGAGTCGGTTGCAGGTCGCTTCTTTTTTA, and NT: 5′-GATCCCCATGGACAGACGCA CGCACGTTCAAGAGACGTGCGTGCGTCTGTCCATTTTTTA-3′. All constructs were confirmed by DNA sequence analysis. The transfection protocol has been previously described^16^. Briefly, 2 × 10^5^ cells were washed twice with PBS and mixed with 0.5 μg of plasmid, and then one electric pulse was applied for 20 ms under a fixed voltage of 1.3 kV on a pipette-type MP-100 microporator (Digital Bio, Seoul, Korea).

### Antisense oligonucleotide

The antisense oligonucleotide (AS) sequence of RSK1: 5′- ATGCCGCTCG**CCCA**GCTCAA-3′ is complementary to the region of the sense template of RSK1. The scramble oligonucleotide (Sc) sequence of RSK1: 5′-GCCCACCCTACGAGCTACGT-3’ was used for the control. Briefly, a final concentration of 100 nM RSK1 antisense oligonucleotide or non-targeting RSK1 scramble oligonucleotide (control) was added in the culture medium for 24 h. The oligonucleotide-treated T47D cells were rendered for quiescent by incubation for 21 h in phenol red free/charcoal-stripped DMEM containing 0.04% FBS. The phenol red free/charcoal-stripped DMEM supplemented with 10% FBS and 0.05% DMSO with or without 50 nM P4 was added to the cell and the culture was conducted for the wound healing assay.

### Statistical analysis

All data were expressed as the mean value±s.e.mean. Comparisons were subjected to one way analysis of variance (ANOVA) followed by Fisher’s least significant difference test. Significance was accepted at *P* < 0.05.

## Additional Information

**How to cite this article**: Wang, H.-C. and Lee, W.-S. Molecular mechanisms underlying progesterone-enhanced breast cancer cell migration. *Sci. Rep.* **6**, 31509; doi: 10.1038/srep31509 (2016).

## Supplementary Material

Supplementary Information
