# Supplementary material for: Molecular mechanisms underlying progesterone-enhanced breast cancer cell migration
Source: Sci Rep. 2016 Aug 11;6:31509. doi: 10.1038/srep31509 (PMC4980668; doi:10.1038/srep31509)
Supplement: Supplementary Information [file srep31509-s1.pdf]

Molecular mechanisms underlying progesterone-enhanced breast cancer cell migration

Hui-Jane Wang[1] and Wen-Sen Lee[1,2,3,*]

1Graduate Institute of Medical Sciences, College of Medicine, Taipei Medical University, Taipei 110, Taiwan

2Department of Physiology, School of Medicine, College of Medicine, Taipei Medical University, Taipei 110, Taiwan

3Cancer Research Center, Taipei Medical University Hospital, Taipei 110, Taiwan

*Corresponding author at: Graduate Institute of Medical Sciences, Taipei Medical University, Taipei 110, Taiwan. Tel: +886-2-2736-1661 ext.3221; Fax: +886-2-2377-8620; E-mail: wslee@tmu.edu.tw

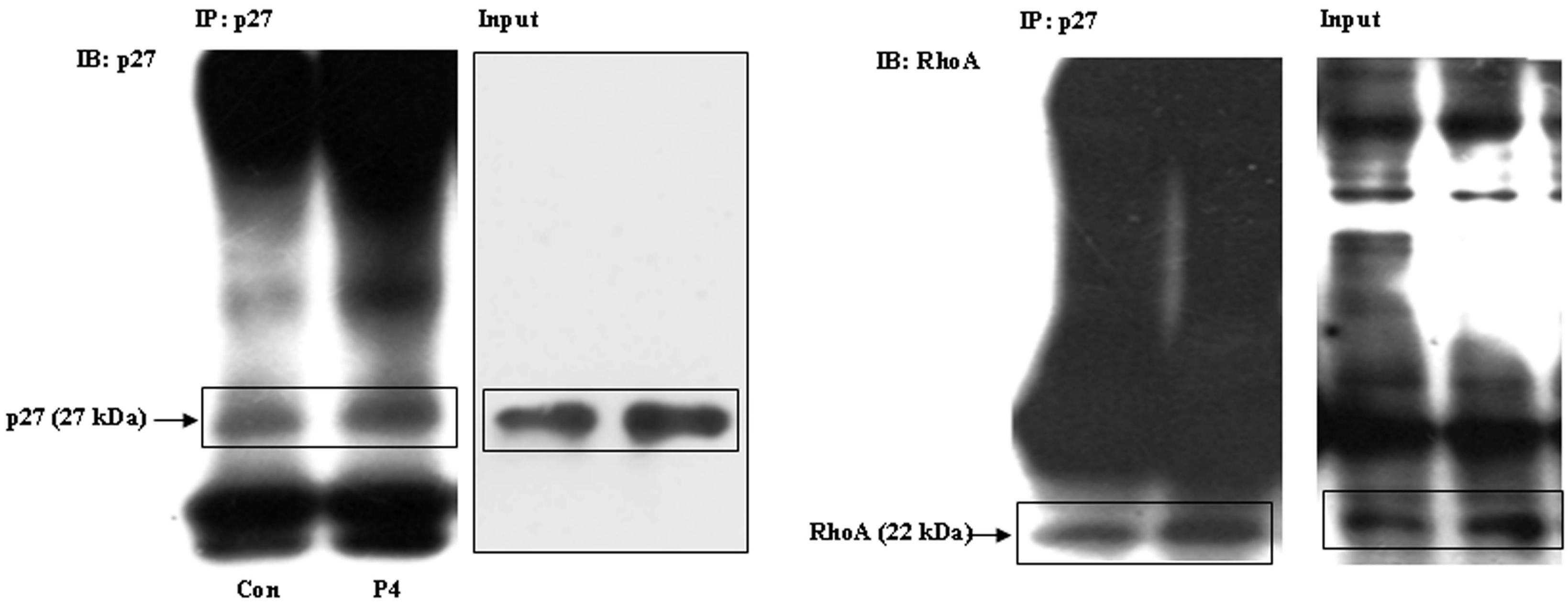

Figure S1. The entire gel pictures of Figure 1C in the text.
